# Heat stress-induced memory impairment is associated with neuroinflammation in mice

**DOI:** 10.1186/s12974-015-0324-6

**Published:** 2015-05-23

**Authors:** Wonil Lee, Minho Moon, Hyo Geun Kim, Tae Hee Lee, Myung Sook Oh

**Affiliations:** Department of Life and Nanopharmaceutical Science, Graduate School and Kyung Hee East–West Pharmaceutical Research Institute, Kyung Hee University, 26 Kyungheedae-ro, Dongdaemun-gu, Seoul, 130-701 Republic of Korea; Molecular Neurobiology Laboratory, McLean Hospital/Harvard Medical School, Belmont, MA 02478 USA; Department of Biochemistry, College of Medicine, Konyang University, Daejeon, 302-718 Republic of Korea; Department of Oriental Pharmaceutical Science, College of Pharmacy, Kyung Hee University, 26 Kyungheedae-ro, Dongdaemun-gu, Seoul, 130-701 Republic of Korea; Department of Formulae Pharmacology, School of Oriental Medicine, Gachon University, 1342 Seongnamdae-ro, Sujeong-gu, Seongnam, 461-701 Republic of Korea

**Keywords:** Heat stress, Thermal stress, Cognition, Neuroinflammation, Synaptic loss, Neuronal death

## Abstract

**Background:**

Heat stress induces many pathophysiological responses and has a profound impact on brain structure. It has been demonstrated that exposure to high temperature induces cognitive impairment in experimental animals and humans. Although the effects of heat stress have long been studied, the mechanisms by which heat stress affects brain structure and cognition not well understood.

**Methods:**

In our longitudinal study of mice exposed to heat over 7, 14, or 42 days, we found that heat stress time dependently impaired cognitive function as determined by Y-maze, passive avoidance, and novel object recognition tests. To elucidate the histological mechanism by which thermal stress inhibited cognitive abilities, we examined heat stress-induced inflammation in the hippocampus.

**Results:**

In mice subjected to heat exposure, we found: 1) an increased number of glial fibrillary acid protein (GFAP)- and macrophage-1 antigen (Mac-1)-positive cells, 2) up-regulated nuclear factor (NF)-κB, a master regulator of inflammation, and 3) marked increases in cyclooxygenase-2 (COX-2), inducible nitric oxide synthase (iNOS), and cytokine interleukin (IL)-1β and tumor necrosis factor (TNF)-α in the mouse hippocampus. We also observed that neuronal and synaptic densities were degenerated significantly in hippocampal regions after heat exposure, as determined by histological analysis of neuronal nuclei (NeuN), postsynaptic density protein 95 (PSD-95), and synaptophysin expression. Moreover, in heat-exposed mice, we found that the number of cells positive for doublecortin (DCX), a marker of neurogenesis, was significantly decreased compared with control mice. Finally, anti-inflammatory agent minocycline inhibited the heat stress-induced cognitive deficits and astogliosis in mice.

**Conclusions:**

Together, these findings suggest that heat stress can lead to activation of glial cells and induction of inflammatory molecules in the hippocampus, which may act as causative factors for memory loss, neuronal death, and impaired adult neurogenesis.

**Electronic supplementary material:**

The online version of this article (doi:10.1186/s12974-015-0324-6) contains supplementary material, which is available to authorized users.

## Background

High environmental temperature is a natural stressor that influences a number of physiological functions and behaviors in animals. Many reports demonstrated that heat stress induces various physiological and pathophysiological responses, such as heat adaptation, hyperthermia, hypoglycemia, water loss, gastric hemorrhage, spermatogenic dysfunction, systemic metabolic disorders, increased blood pressure and heart rate, decreased food consumption and body weight gain, and increased blood glucocorticoid levels and vasopressin release [1–5]. In addition, a number of studies also showed that thermal stress has a profound impact on brain structure and function, leading to neural circuit modification, neuronal loss, neurological defects, convulsions, heat stroke, and accelerated brain dysfunction induced by insults [6–11]. In view of global warming, heat-related diseases have attracted much concern among researchers.

Animal cognitive function plays a critical role in security, guidance, decision making and avoidance of dangerous situations. Stress can result in hippocampal atrophy and cognitive deficits [12, 13]. Moreover, exposure to high temperatures induces cognitive impairment in experimental animals and humans [14, 15]. Hot temperature exposure in humans negatively affects performance in a variety of tasks [16]. Healthy human subjects under passive heat exposure exhibited impaired cognitive ability including perceptive discrimination, short-term memory, and central executive tasks [17, 18]. In experimental animals, heat stress led to slower acquisition and poor retention in a memory task [19]. Although the physiological and behavioral effects of heat stress have long been studied, the mechanisms by which heat stress affects brain structure and cognition are not well understood.

It has been demonstrated that stress contributes to impairment of hippocampus-dependent memory [20]. In addition, stress enhances inflammatory responses [21, 22]; heat stress also increases circulating levels of inflammatory cytokines, such as interleukin (IL)-6 and tumor necrosis factor (TNF)-α in blood [23]. Furthermore, evidence suggests that systemic and central inflammation is directly involved in cognitive decline [24–26]. However, to date, it is unknown whether thermal stress may cause memory deficits associated with neuroinflammation in animals. Therefore, in the present study, we investigated the effects of heat stress on brain inflammation and the mechanisms underlying heat-induced cognitive impairment.

## Methods

### Materials

Rabbit monoclonal anti-glial fibrillary acid protein (GFAP) was purchased from Millipore Bioscience Research (Bedford, MA, USA). Rabbit anti-c-fos and cortisol enzyme-linked immunosorbent assay (ELISA) kits were purchased from Enzo Life Sciences (Farmingdale, NY, USA). Goat anti-heat shock protein 70 (HSP70), anti-doublecortin (DCX), rabbit anti-nuclear factor (NF)-kB, anti-cyclooxygenase-2 (COX-2) and mouse anti-β-actin were purchased from Santa Cruz Biotechnology (Santa Cruz, CA, USA). Rabbit anti-GFAP, mouse anti-neuronal nuclei (NeuN) and anti-inducible nitric oxide synthase (iNOS) were purchased from Millipore Bioscience Research (Bedford, MA, USA). Mouse anti-proliferating cell nuclear antigen (PCNA) was purchased from BD Transduction Laboratories (Franklin Lakes, NJ, USA). Rabbit anti-postsynaptic density protein 95 (PSD-95) and anti-cluster of differentiation molecule 11B (CD11b) were purchased from Abcam (Cambridge, MA, USA). Biotinylated horse anti-goat antibody, goat anti-rabbit antibody, rabbit anti-rat antibody, goat anti-mouse antibody, normal goat serum (NGS), normal horse serum (NHS), and avidin–biotin complex (ABC) were purchased from Vector Labs (Burlingame, CA, USA). Mouse anti-synaptophysin, paraformaldehyde (PFA), 3,3-diaminobenzidine (DAB), sodium chloride, sucrose, ethanol and phosphate buffered saline (PBS) and minocycline hydrochloride were purchased from Sigma–Aldrich (St. Louis, MO, USA). A protein assay kit was purchased from Bio-Rad Laboratories (Hercules, CA, USA). IL-1β and TNF-α ELISA kits were purchased from Ray Biotech (Norcross, GA, USA). The Nuclear/Cytosol Fractionation Kit was purchased from BioVision (Milpitas, CA, USA).

### Animals, heat exposure, and drug treatments

Male imprinting control region (ICR) mice (7 weeks old, 30–32 g) were purchased from the Orient Co., Ltd., a branch of the Charles River Laboratories (Seoul, Korea). The mice were divided randomly into four groups: (1) control, (2) 7-day, (3) 14-day, and (4) 42-day groups. Groups 2–4 were exposed to heat stress once a day for 7, 14, or 42 days, respectively. The animals were housed six per cage (size: 40 cm length, 25 cm width, and 18 cm height) with free access to water and food and were kept under constant temperature (23 ± 1 °C) and humidity (60 ± 10 %) and a 12-h light/dark cycle (lights on at 7:00 a.m. and off at 7:00 p.m.). Animal maintenance and treatments were performed in accordance with the Animal Care and Use Guidelines of Kyung Hee University, Seoul, Korea (approved number; KHP-2014-05-2). Within 1 week of arrival, the mice were adapted to their surroundings for 7 days and kept under the same conditions before the start of the study. To avoid the influence of diurnal cycling, heat exposure began at approximately the same time each day. Heat exposure was achieved by transferring the mice from their home cage into a chamber (Jeio Tech, Daejeon, Korea) maintained at 43 °C and 60 ± 10 % humidity for 15 min once a day. Body weight changes were measured using an electronic balance (OHAUS Corporation, Parsippany, NJ, USA). Body temperature changes were measured using a TC-1000 temperature controller (CWE Inc., Ardmore, PA, USA) inserted into the ear and rectum after terminating heat stress. Mice were subsequently moved back to room temperature. We performed three behavioral tests following the last heat exposure (Additional file 1: Figure S1). For the examination of the anti-inflammatory effect of minocycline, male ICR mice (7-weeks old, 30–32 g) were divided randomly into three groups: (1) normothermic control, (2) heat stress, and (3) heat stress and minocycline treatment. Group (2) and (3) were exposed to high temperature and treated with vehicle (distilled water and saline) and minocycline (50 mg/kg, IP), respectively, once a day for 14 days (Additional file 2: Figure S2).

### Measurement of cortisol levels in serum

A cortisol ELISA was performed according to the manufacturer’s protocol. Briefly, serum was incubated with diethyl ether. The ether mixture was evaporated using nitrogen, after which protease activity was detected using a microplate reader (VERSAmax; Molecular Devices, Sunnyvale, CA, USA), with filters set at 570 nm excitation and 590 nm emission.

### Y-maze test

Alternation rates were assessed using a Y-maze built from black plastic material that had three arms arranged in 120° positions extending from a central space measuring 8 × 8 cm (arm sizes: 30 × 8 × 15 cm). During the 5-min test sessions, each mouse was randomly placed in one arm and allowed to move freely through the maze. Alternation was defined as successive entries into each of the three arms in overlapping triple sets (e.g., A, B, C or B, A, C but not A, B, A). The alternation percentage was calculated as the percentage of actual alternations among the total number of possible arm entries. The arms were cleaned with 70 % ethanol between each test.

### The step-through passive avoidance test

Learning and memory was assessed using a two-compartment step-through passive avoidance apparatus. The box was divided by a guillotine door into bright (21 × 21 × 21 cm) and dark (21 × 21 × 21 cm) compartments. The bright compartment contained an electric lamp, and the floor of the dark compartment was composed of 2-mm stainless steel rods spaced 1 cm apart. The door between the two compartments was opened 10 s later. Then, when the hind legs of the mice entered the dark chamber, the guillotine door was closed, and an electrical foot shock (0.6 mA) was delivered through the grid floor for 3 s. The mice were again placed in the bright chamber for the retention trial, 24 h after the acquisition trial. The time taken for a mouse to enter the dark chamber after the door opening was defined as the latency time, which was recorded for up to 300 s.

### Novel object recognition test

The novel object recognition test was performed according to a previously described method [27, 28]. The experiments were performed in a gray open field box (45 × 45 × 50 cm). Prior to the test, mice were habituated to the test box for 5 min without any objects. After the habituation period, mice were placed into the test box with two identical objects and allowed to explore for 3 min. The objects used in this study were wooden blocks of the same size but of different shapes. The time that the animal spent exploring each object was measured (defined as the familiarization session). Twenty-four hours after the familiarization session, mice were allowed to explore the objects for 3 min, in which a familiar object used in the previous familiarization session was introduced along with a novel object. The time that the animals spent exploring the novel and the familiar objects was recorded (defined as the test session). The animals were considered to be exploring when they were facing, sniffing or biting the object. The test box and objects were cleaned with 70 % ethanol between sessions. Results are expressed as the percentage of the novel object recognition time (time percentage = *t*novel / [*t*novel + *t*familiar] × 100).

### Brain tissue preparation

Two hours after the last heat exposure, mice were immediately anesthetized and perfused transcardially with 0.05 M PBS and then fixed with cold 4 % PFA in 0.1 M phosphate buffer (PB). Brains were removed and post-fixed in 0.1 M PB containing 4 % PFA overnight at 4 °C, and then immersed in a solution containing 30 % sucrose in 0.05 M PBS for cryoprotection. Serial 30-μm-thick coronal sections were cut on a freezing microtome (Leica Instruments GmbH, Nussloch, Germany) and stored in cryoprotectant (25 % ethylene glycol, 25 % glycerol, and 0.05 M PB) at 4 °C until use. For Western blot analysis, mice were decapitated, and the brains were isolated and stored at −80 °C until use.

### Western blot analysis

Hippocampal tissue was lysed using a protein assay kit according to the manufacturer’s instructions. Nuclear proteins were isolated using a Nuclear/Cytosol Fractionation Kit. The lysates were separated by 10 % sodium dodecyl sulfate-polyacrylamide gel electrophoresis (SDS-PAGE), and then transferred to a PVDF membrane (Millipore Bioscience Research). The membranes were incubated with 5 % skim milk in Tris-buffered saline with Tween 20 (TBST) for 1 h and then with a primary antibody (1:1,000; HSP70, c-fos, NF-kB, or iNOS) overnight at 4 °C; this was followed by incubation with a horseradish peroxidase (HRP)-conjugated secondary antibody (Enzo Life Sciences) for 1 h. Immunoreactive-bands were detected using an enhanced chemiluminescence (ECL) detection kit (Bionote, Hwaseong, Korea), and an a LAS-4000 Mini system (Fujifilm Corp., Tokyo, Japan) was used for visualization. Band intensities were normalized to the β-actin band intensity using MultiGauge software (Fujifilm Corp.).

### Immunohistochemistry, Nissl staining and quantification

For immunohistochemical analysis, brain sections were rinsed briefly in PBS and treated with 1 % hydrogen peroxide for 15 min. The sections were incubated with rabbit anti-GFAP, COX-2, NeuN or macrophage-1 antigen (Mac-1; 1:500) and goat anti-DCX antibody (1:250) overnight at 4 °C in the presence of 0.3 % Triton X-100 and NGS. After rinsing in PBS, the sections were then incubated with biotinylated anti-rabbit IgG (1:200) for 90 min and with ABC (1:100) for 1 h at room temperature. Peroxidase activity was visualized by incubating sections with DAB in 0.05 M Tris–buffered saline (TBS, pH 7.6). After several rinses with PBS, sections were mounted on gelatin-coated slides, dehydrated, and coverslipped using Histomount medium (Sigma–Aldrich). For Nissl staining, brain sections were mounted onto slides, stained with 0.5 % Cresyl violet (Sigma-Aldrich), dehydrated through graded alcohol series (70, 80, 90, 95, and 100 %), placed in xylene, and coverslipped using Histomount medium. The images were photographed at 40 × and 100 × magnification using an optical light microscope (Olympus Microscope System BX51; Olympus, Tokyo, Japan) equipped with a 20 × objective lens. The optical density of synaptophysin and PSD-95 in the hippocampus was analyzed using ImageJ software (NIH, USA). To measure optical density, the total region of interest was outlined manually, and the averaged optical densities were acquired in images with converted 8-bit indexed color. The area fractions of GFAP and CD11b/Mac-1 in the hippocampus were measured using ImageJ software. The NeuN- and Nissl-stained cells in CA1 and CA3 pyramidal cell layers were counted under a light microscope and analyzed using ImageJ software. The images were photographed at 40 × and 100 × magnification using an optical light microscope equipped with a 20 × objective lens. Data are presented as percentages of control group values.

### Measurement of IL-1β and TNF-α levels

The mouse IL-1β and TNF-α ELISAs were performed according to the manufacturer’s protocol. Briefly, hippocampal lysates were incubated with reaction buffer. The mixture was incubated for 2.5 h at room temperature before protease activity was detected using a microplate reader, with filters set at 360 nm excitation and 450 nm emission. The samples for each ELISA were run in duplicate, and each ELISA was repeated at least 3 times. The minimum detectable dose of IL-1β and TNF-α is typically less than 5 and 60 pg/ml, respectively, using ELISA kits from Ray Biotech.

### Statistical analysis

All statistical parameters were calculated using Graphpad Prism 5.0 software (Graphpad Software, San Diego, CA, USA). Values are expressed as means ± standard error of the mean (S.E.M.). Statistical comparisons between the different treatments were performed using one-way ANOVA with Tukey’s multiple comparison post test. *p* values of < 0.05 were considered to be statistically significant.

## Results

### The effects of heat exposure on physiological indices for assessing heat stress

Heat exposure reportedly induces physiological, hormonal, and biochemical changes in rodents [1, 29]. To test whether acute and chronic heat exposure induces thermal stress in mice, we examined well-established heat stress indices, such as body weight, body temperature, cortisol level, and heat-shock protein expression in mice. The body weight of heat-stressed groups was lower than that of the normothermic control group, and both rectal and ear temperatures were increased after heat exposure (Additional file 3: Figure S3). Exposure to heat for 3 or 28 consecutive days also increased the level of cortisol in serum significantly (Additional file 4: Figure S4). Next, we performed Western blotting to investigate heat shock protein expression and immediate early gene activation, and found that heat exposure significantly elevated HSP70 and c-fos levels in the mouse hypothalamus (Additional file 5: Figure S5). Consistent with previous reports [1, 29], our data revealed that exposure to high temperature resulted in decreased body weight, increased body temperatures, elevated stress hormones, up-regulated heat shock proteins, and hypothalamic activation, which is responsible for stress responses in the brain. These findings suggest that our experimental conditions (60 ± 10 % humidity at 43 °C for 15 min) are suitable to induce hyperthermia and heat stress in mice.

### The inhibitory effects of heat stress on cognitive behaviors

**Fig. 1:**
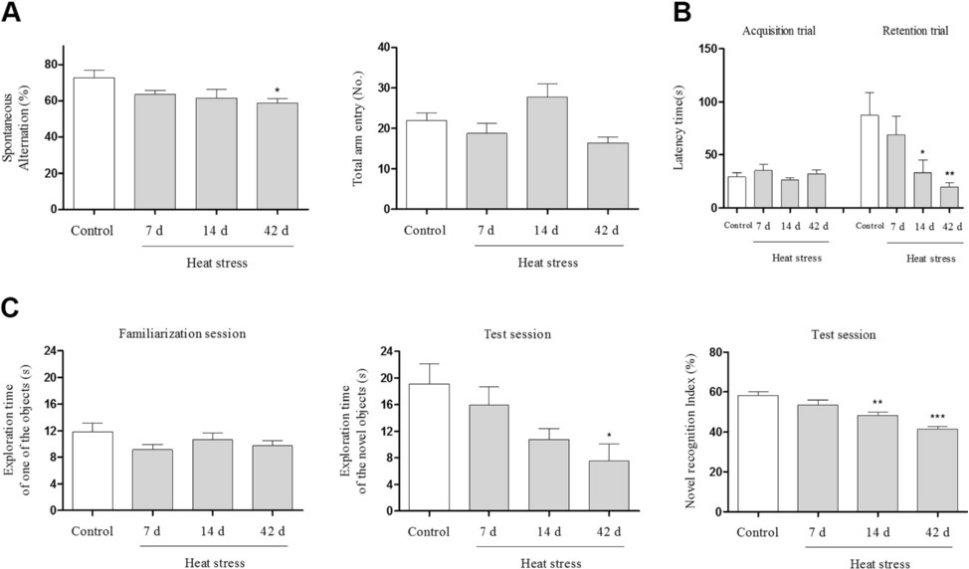
Effects of heat stress on learning and memory in mice. (**a**) After mice (n = 12) were exposed to heat for 7, 14, or 42 days, we determined the percentage of spontaneous alternations and the number of arm entries in the Y-maze. (**b**) Cognitive function was also assessed using a passive avoidance task at different days after heat exposure. Columns represent the latency period for entering the dark box in the acquisition test and in the retention test, 24 h later. (**c**) Memory function after heat stress was determined by a novel object recognition test. Columns indicate the exploration time during the familiarization session and the percentage of time spent near the novel object among the total time spent exploring the objects during the test session. Values are expressed as means ± standard error of the mean (S.E.M.). *p < 0.05, **p < 0.01, and ***p < 0.001 as compared with the control group

Although many researchers have evaluated the relationship between heat and cognition, the inhibitory effect of heat stress on cognitive ability still remains equivocal [14]. Therefore, to determine whether or not heat stress might cause memory loss, we conducted three separate sets of experiments to test learning and memory. First, we assessed spatial working memory by examining spontaneous alternations using a Y-maze task. Exposure time-dependent cognitive impairment was detected in mice subjected to a high temperature (43 °C) for 7, 14, or 42 days (Fig. 1a). We did not observe a significant difference in the total number of arm entries during the Y-maze test in any of the groups (Fig. 1a). As a second memory test, we determined heat-induced cognitive deficits using a passive avoidance test. The retention time of the heat-exposed group was reduced significantly in a heat exposure time-dependent manner (Fig. 1b). No differences were observed in latency time during the acquisition trials among any of the groups (Fig. 1b). Lastly, we utilized a novel object recognition task to examine long-term spatial recognition memory. Control mice spent more time exploring the novel object than the familiar object during the test session. In contrast, heat-exposed mice spent similar amounts of time exploring the novel object and the familiar object during the test session (Fig. 1c). During the familiarization session, no significant differences were found in exploratory preferences among any of the groups (Fig. 1c). These findings clearly demonstrate that heat stress has a time-dependent inhibitory impact on cognitive function in mice.

### The stimulatory effects of heat stress on inflammatory responses in the hippocampus

**Fig. 2:**
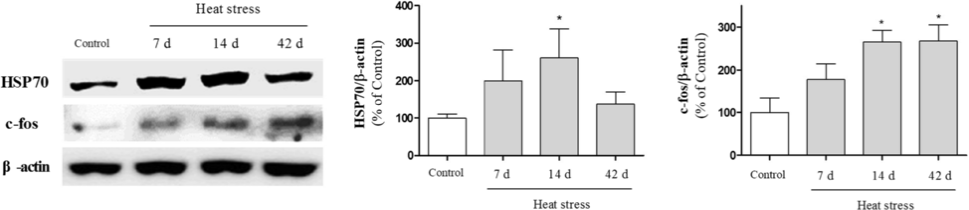
Time-course analysis of heat shock protein 70 (HSP70) and c-fos protein expression in the hippocampus of adult mice following high temperature exposure for 7, 14, or 42 days. Hippocampal lysates were assayed by Western blotting using specific anti-HSP70 and anti-c-fos antibodies. Values are presented as means ± S.E.M. and compared with the control. *p < 0.05 indicates that the mean value is significantly different from the control group

**Fig. 3:**
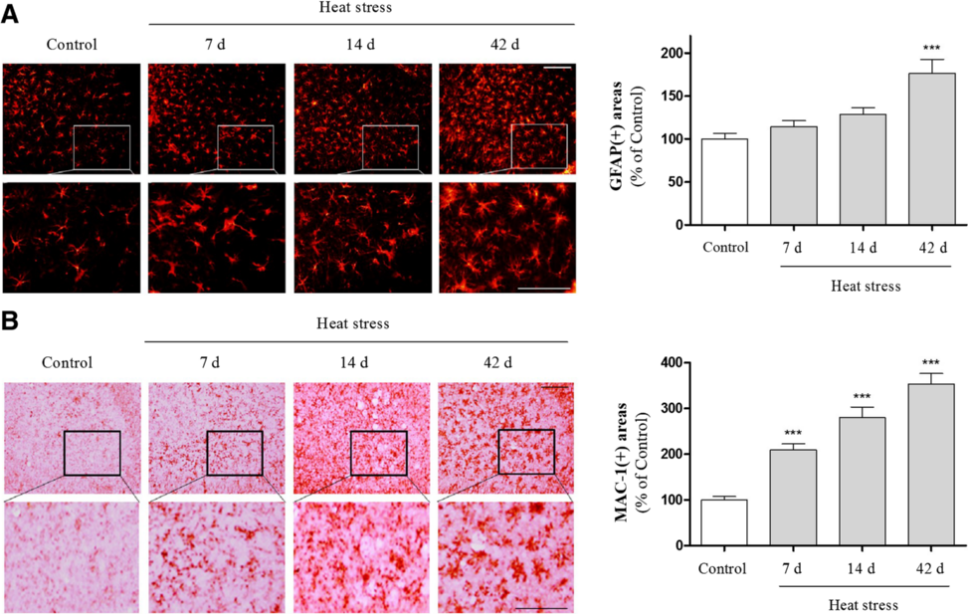
Time-course analysis of heat stress-induced activation of astrocytes and microglia in mouse hippocampus. The presence of astrogliosis (**a**) and microgliosis (**b**) was determined using glial fibrillary acidic protein (GFAP) and macrophage-1 antigen (Mac-1) staining, respectively. Quantification of GFAP- and Mac-1-stained cells was performed by measuring the area fraction of GFAP and Mac-1-immunoreactive cells/areas in the CA3 of the hippocampus. Scale bar = 50 μm. Values are expressed as means ± S.E.M. *** *p* < 0.001 indicates that the mean value was significantly different from the control group

**Fig. 4:**
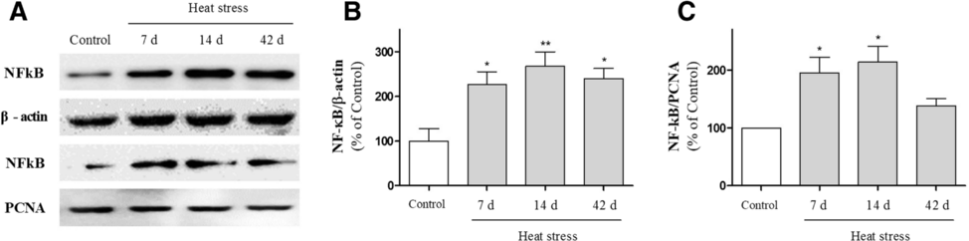
Effects of heat exposure on the nuclear factor (NF)-κB expression in hippocampal whole-tissue lysates and nuclear extracts as measured by Western blot analysis. (**a**) Representative Western blot illustrating the expression of NF-κB in the hippocampus. The graphs display densitometric analyses of the expression ratios of NF-κB/β-actin in whole protein extracts (**b**) and of NF-κB/ proliferating cell nuclear antigen (PCNA) in nuclear extracts (**c**) from the hippocampus. Values are expressed as means ± S.E.M. *p < 0.05 and **p < 0.01 as compared with the control group

It is well established that the hippocampus plays an important role in learning and memory. In addition, stress impairs hippocampus-dependent memory [20], and inflammation is directly related to cognitive deficits [24–26]. Thus, to examine the mechanism by which thermal stress impairs learning and memory, we attempted to detect heat stress-induced inflammation in the hippocampus. We first detected the expression of HSP70 and c-fos in the hippocampus to examine whether heat stress could influence the hippocampus specifically. Western blot analysis showed a marked increase in HSP70 and c-fos expression and peak levels of expression at 14 days following heat stress (Fig. 2). We next investigated the effect of heat stress on glial activation in the hippocampus by immunohistochemistry using antibodies against GFAP and Mac-1. Immunohistochemical analysis revealed that the number of GFAP and Mac-1-stained astrocytes was increased time-dependently in the hippocampus following heat exposure over 7, 14, or 42 days (Fig. 3). Many inflammatory processes are mediated by the activation of NF-κB, a master regulator of inflammation [30]. To confirm heat stress-induced inflammation in the hippocampus, we investigated NF-κB expression using Western blot analysis. NF-κB expression was up-regulated and exhibits peak levels of expression at 14 days in hippocampal whole tissue homogenates (Fig. 4a and b) and nuclear extracts (Fig. 4a and c) of mice subjected to heat stress, suggesting that hyperthermia might induce neuroinflammation in the hippocampus. These results indicate that the memory attenuating effect of heat stress may be mediated by inflammatory responses in the hippocampus.

### The effects of heat stress on expression of inflammatory mediators

**Fig. 5:**
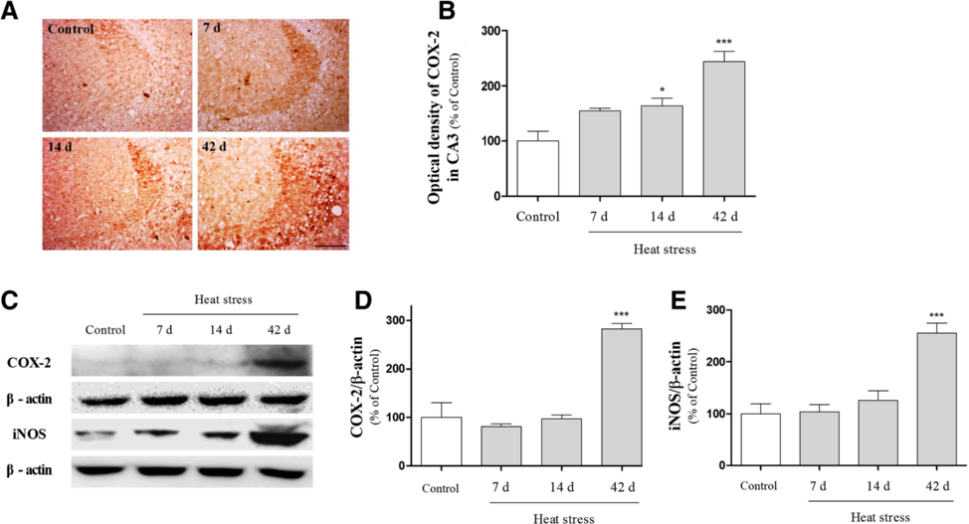
Effects of heat stress on cyclooxygenase 2 (COX-2) and inducible nitric oxide synthase (iNOS) induction in the mouse hippocampus. (**a**) Representative immunohistochemical photomicrographs of COX-2 expression. Scale bar = 50 μm. (**b**) The graphs display the optical density of COX-2 immunoreactivity in the CA3 region. Values are expressed as means ± S.E.M. *p < 0.05 and *** *p* < 0.001 as compared with the control group. (**c**) Representative Western blot showing the expression of COX-2 and iNOS in the hippocampus. The graphs display densitometric analyses of the expression ratios of COX-2/β-actin (**d**) and iNOS/β-actin (**e**) in the mouse hippocampus. Values are expressed as means ± S.E.M. *** *p* < 0.001 as compared with the control group

**Fig. 6:**
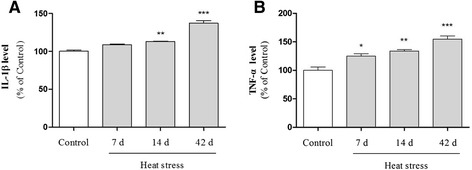
Effects of heat stress on IL-1β and TNF-α production in the mouse hippocampus. Male imprinting control region (ICR) mice were exposed to high temperature for 7, 14, or 42 days. Expression of IL-1β (**a**) and TNF-α (**b**) was assessed using a sandwich enzyme-linked immunosorbent assay (ELISA). Values are expressed as means ± S.E.M. *p < 0.05, **p < 0.01, and ***p < 0.001 as compared with the control group

NF-κB acts as an essential transcription factor for the induction of inflammatory mediators, such as iNOS, COX-2, and cytokines [30]. In addition, proinflammatory molecules have been proposed to inhibit the induction of long-term potentiation (LTP) and contribute to the cognitive decline. To examine the effect of heat stress on induction of the proinflammatory mediators COX-2 and iNOS, we performed immunohistochemical and Western blot analyses. Expression of COX-2 and iNOS was time-dependently up-regulated in the mouse hippocampus after heat exposure (Fig. 5). Using ELISA, we then investigated whether thermal stress led to the release of cytokines, such as IL-1β and TNF-α in the hippocampus. Our data revealed that IL-1β and TNF-α production in the hippocampus of mice subjected to heat stress for 7, 14, and 42 days was significantly higher than that in the control mice (Fig. 6). Thus, these proinflammatory mediators were increased markedly in the hippocampus due to heat stress.

### The deleterious effects of heat stress on brain structure

**Fig. 7:**
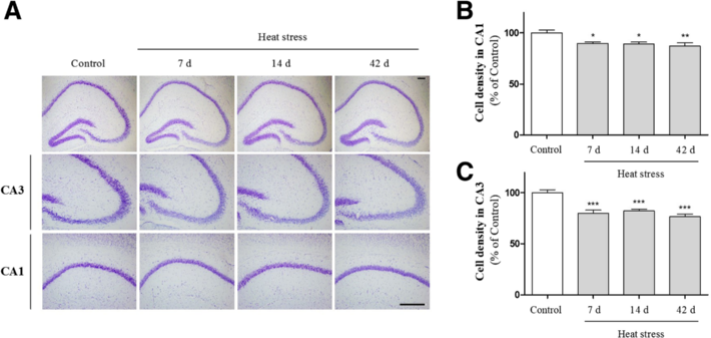
Effects of heat stress on cell death in the hippocampus. (**a**) Representative pictures of Cresyl violet staining in the granule cell layer and the pyramidal cell layer of the hippocampus. Cresyl violet-stained cells were markedly lower in the heat-treated groups than the control group. The number of cells was reduced significantly in both the CA1 (**b**) and CA3 (**c**) regions. Values are expressed as means ± S.E.M. *p < 0.05, **p < 0.01, and ***p < 0.001 as compared with the control group

**Fig. 8:**
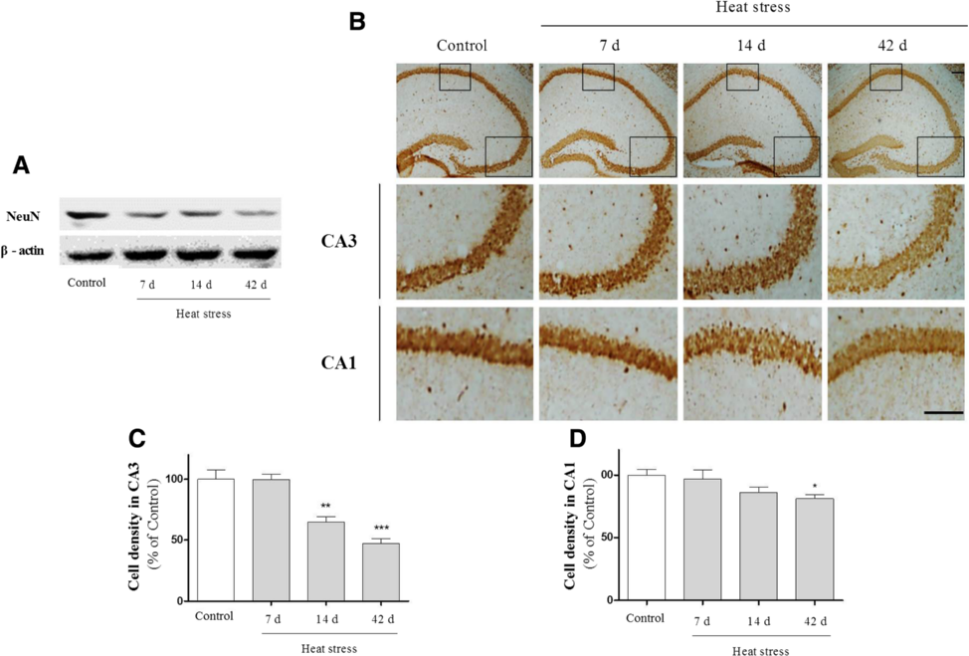
Effects of heat stress on neuronal loss in the hippocampus. (**a**) Representative Western blot showing the expression of neuronal nuclei (NeuN), a marker for neuronal cells, in the hippocampus. (**b**) Representative pictures of NeuN staining in the granule cell layer and the pyramidal cell layer of the hippocampus. NeuN-stained cells were markedly lower in the heat-treated groups than the control group. The number of cells was reduced significantly in both the CA3 (**c**) and CA1 (**d**) regions. Values are expressed as means ± S.E.M. *p < 0.05, **p < 0.01, and ***p < 0.001 as compared with the control group

**Fig. 9:**
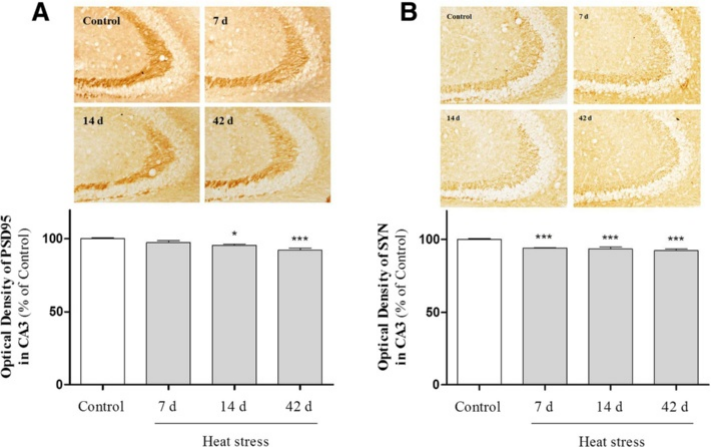
Effects of heat stress on synaptic density in the hippocampus. Representative photomicrographs of postsynaptic density protein (PSD)-95 and synaptophysin immunohistochemistry in CA3. Note that compared with control mice, high temperature-exposed mice exhibit decreased PSD-95 and synaptophysin immunoreactivity. Quantification of the average intensity of the immunostaining of (**a**) PSD-95 and (**b**) synaptophysin in the CA3. *p < 0.05 versus the control group. Scale bar = 50 μm

Memory deficits strongly correlate with synaptic protein expression and hippocampal synaptic density; synaptic damage is associated directly with neuroinflammation [31–34]. Heat stress reportedly induces neuronal loss in rat hippocampus [35, 36]. However, no evidence exists suggesting that heat exposure might lead to synaptic loss in the hippocampus. First, we determined whether or not heat stress resulted in hippocampal neuronal loss using Nissl staining and NeuN immunostaining. Nissl- and NeuN-stained coronal brain sections showed that heat stress significantly reduced neuronal density in the CA1 and CA3 pyramidal cell layers (Figs. 7 and 8b-d). Some cells exhibited weak staining, suggesting that they were in the process of degenerating. Western blot analysis using an antibody recognizing the NeuN neuronal marker also revealed that heat stress for 7, 14, and 42 days induced neuronal loss in the hippocampus (Fig. 8a). Next, to investigate the effects of heat stress on synaptic density, we examined the expression of synaptic marker molecules, such as PSD-95 and synaptophysin, in the hippocampus. Optical densitometry data showed that both PSD-95 and synaptophysin immunoreactivity were time-dependently decreased in the CA3 subfields of the hippocampus in heat-exposed mice compared with control mice (Fig. 9). For the first time, we demonstrated that thermal stress might lead to synaptic damage in the hippocampus.

### The effects of heat stress on neurogenesis

**Fig. 10:**
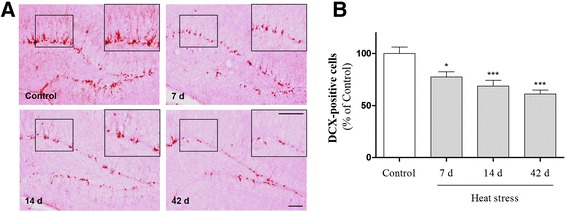
The effects of heat stress on adult neurogenesis in the hippocampus. (**a**) Representative images of doublecortin (DCX)-stained cells in the dentate gyrus. Scale bar = 50 μm. (**b**) Quantification of the number of DCX-expressing cells in the subgranular zone (SGZ). The cell number was decreased significantly in heat-exposed mice compared with control mice. *p < 0.05 and ***p < 0.001 as compared with the control group

It has been proposed that some cognitive impairment related to neuroinflammation might be associated with inflammation-induced deficits in adult hippocampal neurogenesis [37, 38]. Additionally, inflammatory molecules released from activated glial cells may inhibit the generation of functional neurons from adult neural stem cells [39]. Therefore, to investigate whether inflammation-stimulated heat stress can induce deficits in adult neurogenesis in the hippocampus, we examined the expression level of an adult neurogenesis marker in mice subjected to heat stress. DCX is transiently induced by immature granule cells, and the detection of DCX protein for neurogenesis analysis does not require *in vivo* labeling of proliferating cells [40, 41]. Mice exposed to heat stress over 7, 14, and 42 days showed a time-related reduction in the number of DCX-positive cells in the subgranular zone (SGZ) compared with control mice (Fig. 10). These results suggest that heat stress may have an inhibitory effect on adult hippocampal neurogenesis by acting as an inflammation-inducing factor.

### The inhibitory effects of anti-inflammatory agent on cognitive deficit and neuroinflammation induced by heat-stress

**Fig. 11:**
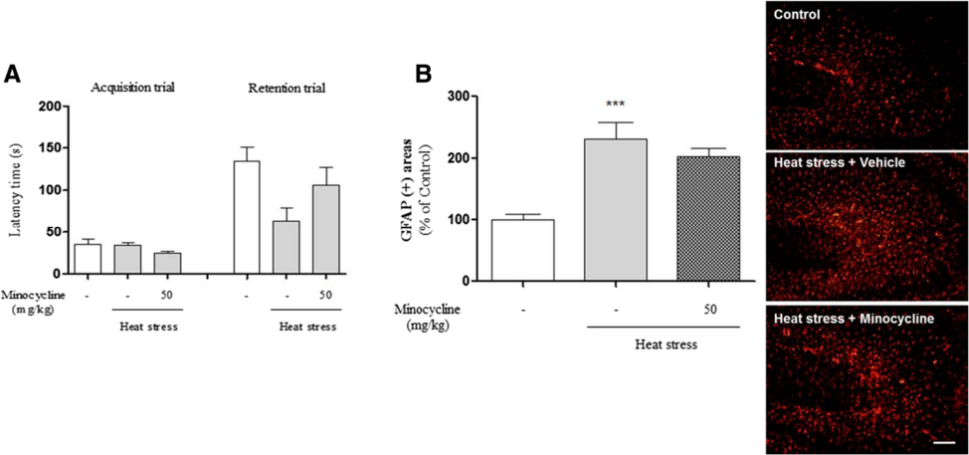
Effects of anti-inflammatory agent on heat-stress-induced memory loss and astrogliosis. (**a**) Cognitive function was assessed using a passive avoidance task after heat exposure and minocycline treatment. Columns represent the latency period for entering the dark box in the acquisition test and in the retention test, 24 h later. (**b**) The astrogliosis was analyzed and determined using glial fibrillary acidic protein (GFAP) staining. Quantification of GFAP-stained cells was performed by measuring the area fraction of GFAP-immunoreactive cells/areas in the CA3 of the hippocampus. Scale bar = 50 μm. Values are expressed as means ± S.E.M. *** *p* < 0.001 indicates that the mean value was significantly different from the control group

To clearly examine the relationship with inflammation and cognitive function, we treated heat-stressed animals with anti-inflammatory agent minocycline. We determined heat-induced cognitive deficits and minocycline-induced inhibition of memory loss using a passive avoidance test. The retention time of the heat-exposed and minocycline-treated group was increased compared with heat-exposed and vehicle-treated group (Fig. 11a). No differences were observed in latency time during the acquisition trials among any of the groups (Fig. 11a). Next, we investigated the effect of minocycline on heat stress-induced glial activation in the hippocampus by immunohistochemistry using antibody against GFAP. Immunohistochemical analysis revealed that the number of GFAP-stained astrocytes was significantly decreased in the hippocampus following heat exposure and minocycline treatment, compared with heat-exposure and vehicle-treatment (Fig. 11b). Our results suggest that heat stress-induced memory loss can be mediated by neuroinflammation in the hippocampus.

## Discussion

In the present study, we demonstrated that heat stress significantly impaired learning and memory in mice. To examine the histological mechanisms underlying the memory-inhibiting action of heat stress, we investigated the degree of neuroinflammation, neuronal and synaptic loss, and adult neurogenesis in the hippocampus of mice subjected to heat exposure. Thermal stress (1) induced the activation of glial cells and proinflammatory molecules, (2) resulted in neuronal and synaptic loss, and (3) led to abnormal adult neurogenesis in the hippocampus. Our findings suggest that heat stress might impair cognitive function by leading to neuroinflammation, neurodegeneration, and defective neurogenesis in the hippocampus.

Although there are a substantial number of studies regarding physiological responses to thermal stress, effects on memory function still remain controversial. Differences in experimental methods among studies have made it difficult to interpret whether or not exposure to heat per se may contribute to cognitive deficits [14]. To clearly elucidate the heat-cognition relationship, we examined the dependency on heat exposure time. Based on a previous report [6, 14], we chose the following experimental methods and conditions: humidity (60 ± 10 %), temperature (43 °C), exposure duration (15 min/day), subject number (n = 12), and memory test paradigms (Y-maze, novel object recognition task, and passive avoidance test). By conducting a longitudinal study, we clearly demonstrated that heat exposure per se had an inhibitory effect on cognition in mice.

Glial cells have a critical role in inflammatory responses in the central nervous system (CNS) and are activated during neuropathological conditions [42]. Glial activation involves proliferation, migration to the damaged regions, and induction of proinflammatory molecules, such as cytokines, COX-2 and iNOS [42, 43]. In addition, the activated glia can result in neuronal and synaptic injury through the release of proinflammatory and cytotoxic factors, including iNOS-derived nitric oxide, COX-2-derived prostaglandin E_2_, TNF-α, and IL-1β [42, 44]. Furthermore, the TNF-α, and IL-1β released from activated glial cells may inhibit the proliferation of adult neural stem cell and cognitive functions [24, 39, 45].

It has been reported that stress may influence systemic inflammatory responses [21, 22]: thermal stress enhances circulating levels of inflammatory cytokines, such as IL-6 and TNF-α in blood [23]. However, the effects of heat stress on brain inflammation and the mechanisms underlying heat-induced cognitive impairment are unknown to date. The expression of genes and proteins associated with learning and memory precedes the behavioral changes. NF-κB has been known to act as an essential transcription factor for the induction of inflammatory mediators, such as iNOS, COX-2, IL-1β and TNF-α [46]. In addition, the NF-κB signaling may play a most critical role in activation of glia cells [47], indicating that the activation of NF-κB signaling precedes the microglial and astroglial activation. Furthermore, the proinflammatory molecules and activated glial cells have been shown to contribute to the cognitive decline [24–26]. Taken together, our data suggest that heat stress might induce activation of NF-kB, which subsequently leads to the elevation of TNF-α and IL-1β, and the up-regulation of iNOS and COX-2, resulting in cognitive deficits in mice.

Accumulating experimental evidence supports the concept that high temperature exposure results in memory-reducing effects in humans [18, 48, 49]. Some studies suggested mechanisms regarding the impairment of cognition by heat stress: increased body temperature after heat exposure might increase the general level of arousal in subjects, resulting in insufficient attention to the learning task [19]. However, the mechanisms involved in the negative effect of heat exposure on cognitive function remain unclear. For the first time, we found that heat-exposed mice exhibited a marked increase in hippocampal neuroinflammation. In addition, it is well established that inflammatory responses can directly contribute to cognitive impairment [45]. Thus, heat-induced neuroinflammatory responses in the hippocampus may be one of the mechanisms involved in the action of heat stress that subsequently results in memory deficits.

A number of studies showed that heat stress can cause cell death in the brain [6, 35, 36, 50]. Activated glial cells in the brain produce neurotoxic factors that subsequently lead to neuronal death [44]. However, there is no evidence regarding the relationship between neurodegeneration and neuroinflammation under heat stress. In this study, we found that high temperature exposure significantly influenced both neuroinflammatory responses and neuronal cell death in the mouse hippocampus. Moreover, there are no reports to date on the mechanism by which heat stress affects synaptic loss. Thus, to investigate the influence of thermal stress on synaptic density *in vivo*, we assessed the expression of PSD-95, a postsynaptic density marker, and synaptophysin, a presynaptic density marker, using immunostaining in the hippocampal region of mice exposed to heat stress. Immunoreactivity of synaptic molecules was decreased significantly in mice exposed to high temperature (Fig. 9). For the first time, we demonstrated that heat stress caused synaptic degeneration in the hippocampus. Taken together, it is possible that the neurodegenerative effects of heat stress may be caused by a glia-activating action.

It is not known whether heat stress impacts adult hippocampal neurogenesis. Our results showed that cognitive impairment due to heat stress was accompanied by deficits in adult neurogenesis (Fig. 10). It is widely believed that adult neurogenesis in the hippocampus contributes to learning and memory [51]. Based on these findings, we speculate that heat stress-induced production of toxic factors from glial cells may inhibit adult neurogenesis, resulting in accelerated cognitive impairment by heat exposure. Our data suggest that cognitive impairment due to heat stress may be associated with impaired neurogenesis in the hippocampus of adult mice.

In summary, we found that mice exhibit cognitive impairment, neurodegeneration, and defective neurogenesis after exposure to high temperature. Furthermore, heat stress leads to glial cell activation and induction of inflammatory molecules in the hippocampus, which are well-known causative factors of memory loss, neuronal death, and impaired adult neurogenesis. Taken together, targeting neuroinflammation could be a therapeutic strategy for the treatment of heat-related cognitive deficits and diseases.

## Additional files

Additional file 1: Figure S1. Timeline representing the duration of heat exposure. A diagram of the minocycline treatment method is shown. Before the last heat exposure period, mice were administered minocycline (50 mg/kg) for 14 days. Additional file 2: Figure S2. Timeline representing the duration of heat exposure and minocycline treatment. PAT: passive avoidance test. Additional file 3: Figure S3. The effects of heat exposure on body weight and temperature. Heat stress groups were exposed to heat (43 °C for 15 min once daily) for 3 days (A) or 4 weeks (B). Additional file 4: Figure S4. The effects of acute (3 days) and chronic (28 days) heat exposure on cortisol levels in mouse serum. Cortisol expression in serum was assessed using a sandwich enzyme-linked immunosorbent assay (ELISA) after heat stress for 3 days (A) or 28 days (B). Values are presented as means ± standard error of the mean (S.E.M.) and compared with day 0. ***p* < 0.01 indicates that the mean value was significantly different from the control group value. Additional file 5: Figure S5. Effects of acute and chronic heat exposure on the expression of heat shock protein 70 (HSP70) and c-fos in the hypothalamus. After mice (n = 12) were exposed to heat for 3 days (A) or 28 days (B), the hypothalamus was dissociated and lysed to measure HSP70 and c-fos expression using Western blotting. Values are presented as means ± S.E.M. and compared with normothermic controls. **p* < 0.05 and ***p* < 0.01 indicate that the mean value was significantly different from the control group.
