# Supplementary figures and images for: Heat stress-induced memory impairment is associated with neuroinflammation in mice
Source: J Neuroinflammation. 2015 May 23;12:102. doi: 10.1186/s12974-015-0324-6 (PMC4465309; doi:10.1186/s12974-015-0324-6)

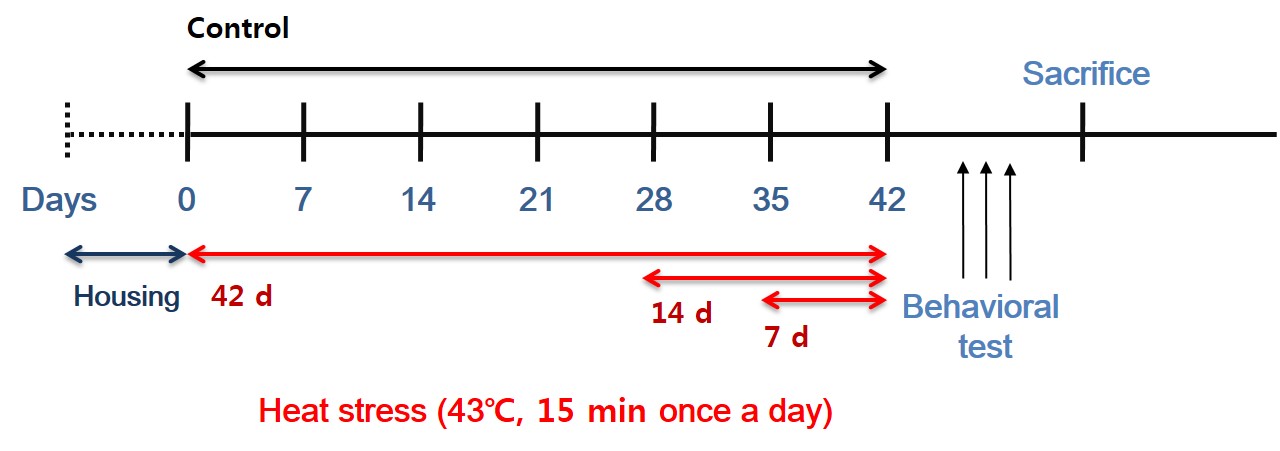

Supplement: Additional file 1: Figure S1. — Timeline representing the duration of heat exposure. A diagram of the minocycline treatment method is shown. Before the last heat exposure period, mice were administered minocycline (50 mg/kg) for 14 days. [file 12974_2015_324_MOESM1_ESM.jpeg]

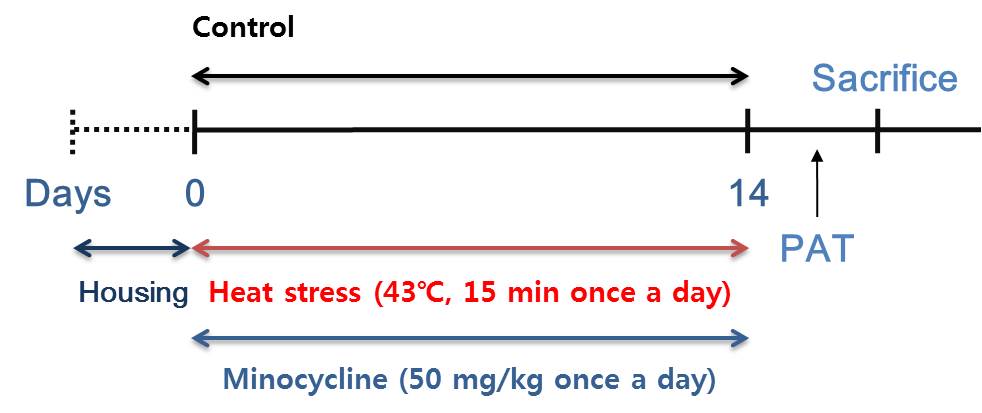

Supplement: Additional file 2: Figure S2. — Timeline representing the duration of heat exposure and minocycline treatment. PAT: passive avoidance test. [file 12974_2015_324_MOESM2_ESM.jpeg]

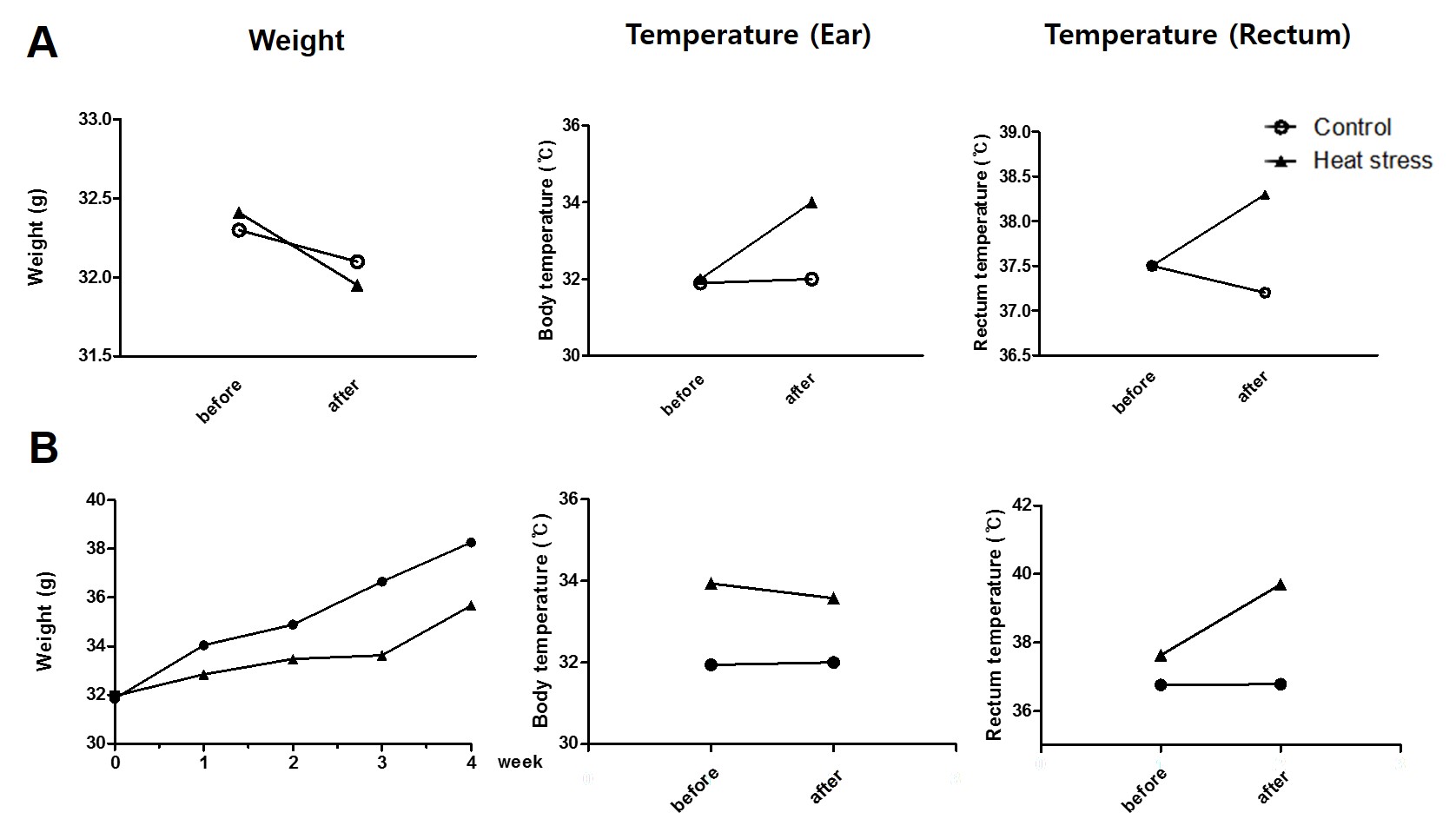

Supplement: Additional file 3: Figure S3. — The effects of heat exposure on body weight and temperature. Heat stress groups were exposed to heat (43 °C for 15 min once daily) for 3 days (A) or 4 weeks (B). [file 12974_2015_324_MOESM3_ESM.jpeg]

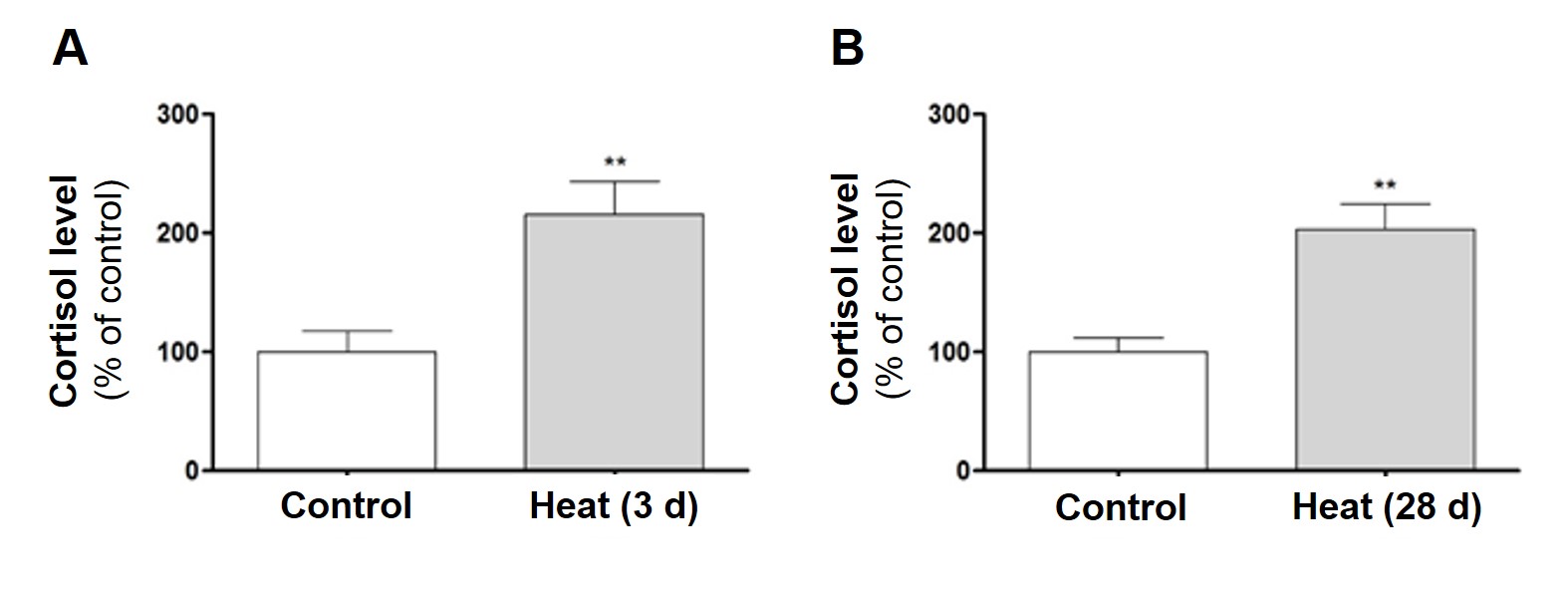

Supplement: Additional file 4: Figure S4. — The effects of acute (3 days) and chronic (28 days) heat exposure on cortisol levels in mouse serum. Cortisol expression in serum was assessed using a sandwich enzyme-linked immunosorbent assay (ELISA) after heat stress for 3 days (A) or 28 days (B). Values are presented as means ± standard error of the mean (S.E.M.) and compared with day 0. **p < 0.01 indicates that the mean value was significantly different from the control group value. [file 12974_2015_324_MOESM4_ESM.jpeg]

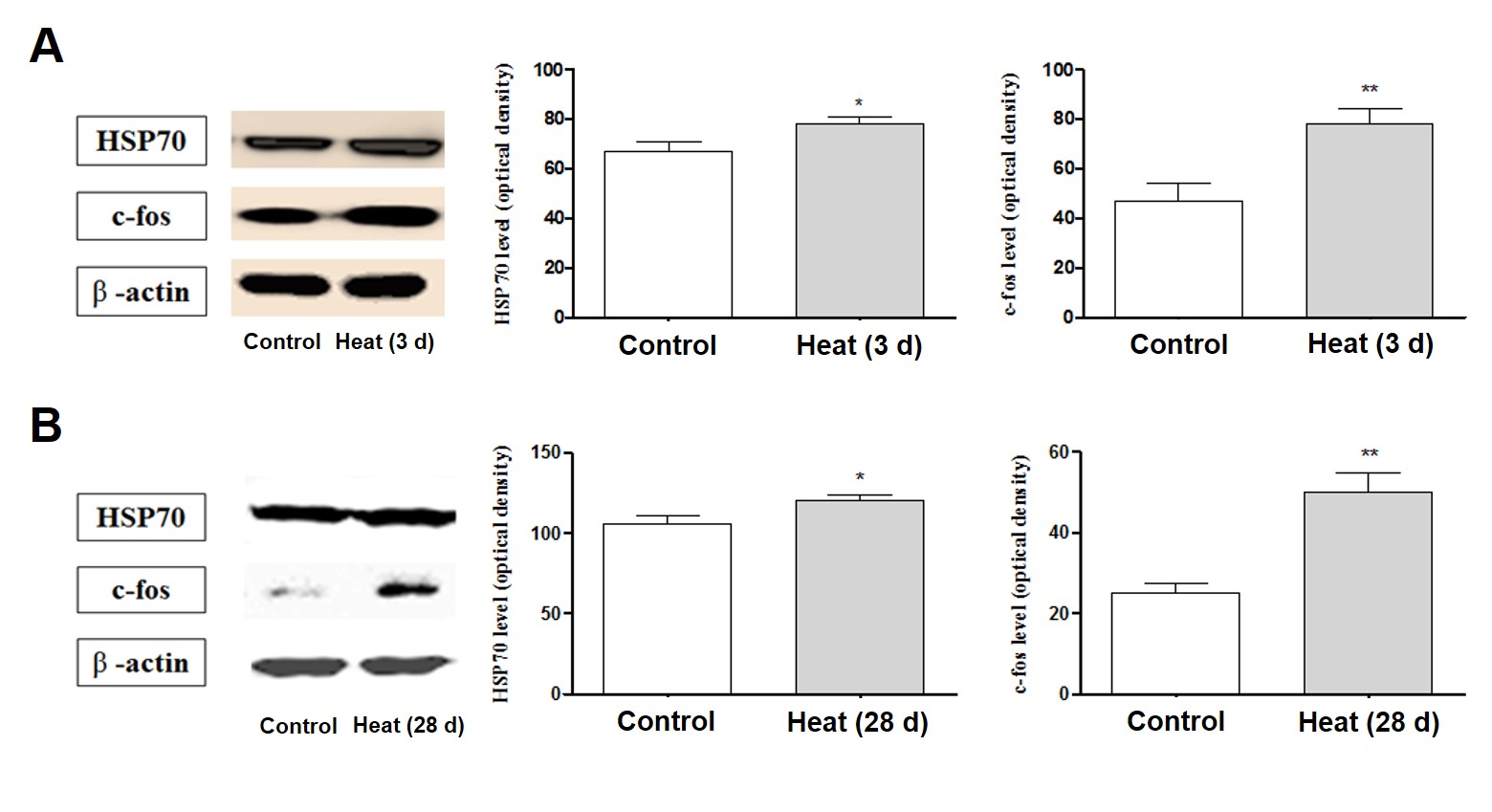

Supplement: Additional file 5: Figure S5. — Effects of acute and chronic heat exposure on the expression of heat shock protein 70 (HSP70) and c-fos in the hypothalamus. After mice (n = 12) were exposed to heat for 3 days (A) or 28 days (B), the hypothalamus was dissociated and lysed to measure HSP70 and c-fos expression using Western blotting. Values are presented as means ± S.E.M. and compared with normothermic controls. *p < 0.05 and **p < 0.01 indicate that the mean value was significantly different from the control group. [file 12974_2015_324_MOESM5_ESM.jpeg]
